# Whole-Genome Sequence of *Mycobacterium abscessus* Clinical Strain V06705

**DOI:** 10.1128/genomeA.00690-13

**Published:** 2013-09-05

**Authors:** Stanley Pang, Aurélie Renvoisé, Claire Perret, Marie Guinier, Nadjim Chelghoum, Florence Brossier, Estelle Capton, Vincent Jarlier, Wladimir Sougakoff

**Affiliations:** Laboratoire de Bactériologie-Hygiène, ER-5, Université Pierre et Marie Curie, UPMC, Paris, Francea; Génétique Épidémiologique et Moléculaire des Pathologies Cardiovasculaires, UMR_S 937, Paris, Franceb; IFR14—P3S, Université Pierre et Marie Curie—Paris, Paris, Francec

## Abstract

Infection caused by *Mycobacterium abscessus* strains is a growing cause of concern in both community-acquired and health care-associated diseases, as these organisms naturally display multiple drug resistances. We report an annotated draft genome sequence of *M. abscessus* strain V06705 obtained from a patient in France.

## GENOME ANNOUNCEMENT

Nonpigmented rapidly growing mycobacteria are ubiquitous environmental microorganisms. *Mycobacterium abscessus* was previously divided into three species (*M. abscessus sensu stricto*, *Mycobacterium massiliense*, and *Mycobacterium bolletii*), but in 2009, Leao et al. (1) proposed the union of *M. bolletii* and *M. massiliense*, and also the recognition of two subspecies within *M. abscessus*: *M. abscessus* subsp. *abscessus* and *M. abscessus* subsp. *bolletii*. *M. abscessus* is an emerging human pathogen responsible for a wide spectrum of soft tissue infections (which includes nosocomial diseases) and disseminated infection in immunocompromised patients, and it is a major respiratory pathogen, particularly in individuals with cystic fibrosis or chronic pulmonary disease (2, 3). Treatment of these infections involves a combination of antibiotics but is challenging due to extended intrinsic and acquired resistances to aminoglycosides and macrolides (4).

Prior genomic analyses of *M. abscessus* genomes have raised further questions on the method by which antibiotic resistance is conferred, whether it is introduced by transmissible genetic elements (5) or spontaneous mutations (4). Recently, spontaneous mutations found in genes encoding antibiotic targets have been found to give rise to resistance (6).

We present the draft genome sequence of *M. abscessus* strain V06705, which was isolated from a patient bronchoalveolar lavage sample. The strain was shotgun sequenced on an Illumina HiSeq 2000 (Illumina, Inc.). Illumina sequences were assembled using Velvet v1.2.9 (7), resulting in 90 contigs and an N_50_ contig size of 174,031 bp. The draft genome sequence is 5,264,933 bp in length based on 7,720,556 reads, with a G+C content of 64.9%. The genome sequence was annotated using the NCBI Prokaryotic Genomes Automatic Annotation Pipeline (http://www.ncbi.nlm.nih.gov/genomes/static/Pipeline.html). The automated annotation identified 5,129 coding DNA sequences with 46 tRNA- and 3 rRNA-encoding genes. Genomic comparisons between isolate V06705 and the reference strain *M. abscessus* ATCC 19977 (CIP 104536^T^) (8) show greater sequence homology than comparisons with other *M. abscessus* genomes (9). ATCC 19977 is a rough strain that is hyperlethal to mice and contains the loss in function required to produce glycopeptidolipids (10). Therefore, the genome sequence of V06075 makes an ideal candidate for identifying other regions containing high rates of mutation and transmissible genetic elements that contribute to virulence.

### Nucleotide sequence accession number.

The *M. abscessus* strain V06705 genome sequence and annotation data have been deposited in NCBI GenBank under the accession no. AUMY00000000.
